# Isolated Celiac and Splenic Artery Dissection: A Case Report and Review of the Literature

**DOI:** 10.1155/2015/194079

**Published:** 2015-12-24

**Authors:** Tania Moussa, Georges Nawfal, Tarek Assi, Elie El Rassy, Elie Massoud, Iskandar Daou

**Affiliations:** ^1^Department of Radiology, Hotel Dieu de France University Hospital, Faculty of Medicine, Saint Joseph University, Beirut 1104 2020, Lebanon; ^2^Department of Radiology, Saint Joseph University Hospital, Faculty of Medicine, Saint Joseph University, Beirut 90375, Lebanon; ^3^Department of Hematology-Oncology, Hotel Dieu de France University Hospital, Faculty of Medicine, Saint Joseph University, Beirut 1104 2020, Lebanon; ^4^Department of Vascular Surgery, Saint Joseph University Hospital, Faculty of Medicine, Saint Joseph University, Beirut 90375, Lebanon

## Abstract

An isolated dissection of the celiac artery is an extremely rare condition that requires a high level of suspicion to evoke the diagnosis. Once established, the natural course is unpredictable in view of the discrepancies in its management requiring a case-by-case analysis. In this paper, we report an unusual case of spontaneous abdominal pain that was diagnosed with celiac and splenic artery rupture secondary to physical stress. This paper underlines the necessity to maintain a high level of suspicion for arterial dissections and we also review the management plan in such cases.

## 1. Introduction

Arterial dissections are not rare findings in the emergency departments that are often diagnosed in a context of predisposing risk factors [1]. Spontaneous isolated celiac artery dissection (SICAD) is one possible alarming etiology that is often undermined in the emergency department [1, 2]. To our knowledge, the very few cases reported of SICAD are spontaneous without an established causative factor. However, albeit a high level of suspicion may be of benefit, the natural course of the disease is unpredictable. Effectively, the optimal management plan remains largely controversial. In this paper, we report an unusual case of SICAD secondary to physical stress that fully recuperated the arterial permeability after adequate anticoagulation.

## 2. Case Report

**Figure 1:**
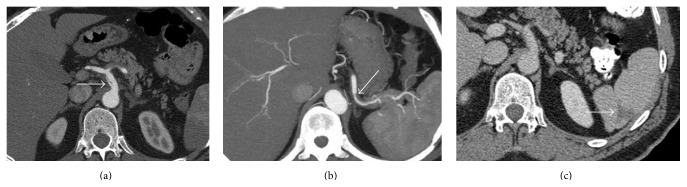
Axial enhanced CT scan shows (a) an intimal dissection of the celiac artery with opacification of true and false lumen, (b) a dissection membrane in the splenic artery causing poor contrast flow and thrombosis of the false lumen, and (c) a posterolateral splenic infarction.

**Figure 2:**
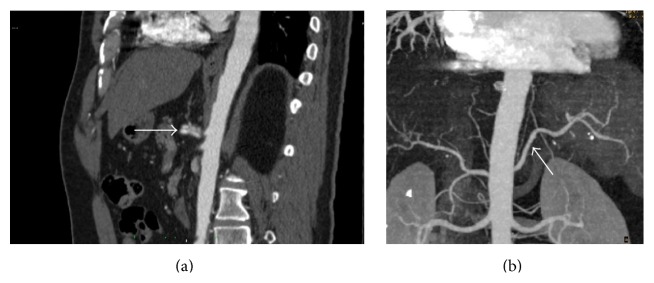
Control CT scan after one month. (a) Sagittal contrast-enhanced CT scan shows ectasia of the celiac artery and stability of intimal dissection compared to the previous scan. (b) 3D reconstruction of the aorta and branch vessels shows patent splenic artery.

An otherwise healthy 50-year-old man presented to our emergency department for acute onset of isolated diffuse sharp abdominal pain that occurred while jogging at the gym. His medical history is only relevant for a one-week upper respiratory viral infection treated symptomatically. Clinical exam was strictly normal except for a blood pressure of 170/90 mmHg. Blood tests showed normal blood count and chemistry. D-dimers were negative. Activated partial thromboplastin time and prothrombin time were normal (37 seconds and 12 seconds, resp.). Abdominal computed tomography angiogram (CTA) revealed a celiac trunk dissection extending to the splenic artery. The hepatic, superior mesenteric, and aortic artery showed normal features but stigmata of splenic infarction were noted with partial thrombosis at the splenic hilum (Figure 1). Investigations did not reveal associated connective tissue disease. The patient was managed by analgesics and subcutaneous enoxaparin 60 mg BID shifted after 72 h of bridging to Coumadin anticoagulation therapy for six months for an INR 2-3. Follow-up CT scan performed one month later showed a stable celiac artery dissection with repermeabilisation of the splenic artery (Figure 2).

## 3. Discussion

Arterial dissection is a disruption of subintimal-medial or the medial-adventitial layers of vessels. The most common site for arterial dissection is renal arteries followed by intracranial, coronary, and pulmonary arteries [1]. SICAD is an undermined and alarming condition that requires a high level of suspicion to prevent a fatal prognosis [1, 2]. It occurs most commonly in males with a median age of 55 years at diagnosis [3]. Patients are often asymptomatic but symptoms are often atypical with abdominal pain, pancreatitis, malabsorption, intestinal angina, and jaundice [4, 5].

The majority of celiac artery dissections are idiopathic but occur with predisposing factors of atherosclerosis, pregnancy, trauma, and connective tissue disease [6]. Infections are also another risk factor for spontaneous arterial dissections. This has been suggested secondary to observational studies reporting increased incidence of such dissections with seasonal variations, especially in autumn [7]. Exertion and sudden abdominal hyperpressure (sneezing, lifting, or even Valsalva maneuver) are possible precipitating factors via mechanical stretching and microtrauma on the arterial wall. The exact pathophysiologic mechanism is not clear yet but it is usually attributed to extreme variations in both diastolic and systolic blood pressure [8]. This pressure pattern is considerable in weightlifters but SCIAD is uncommon and only once reported in the literature [9–11]. Our patient did not present clear risk factors for SCIAD; we considered a culminating effect of the viral infection and exertion (jogging) to induce the SCIAD in an otherwise healthy person.

Diagnostic imaging relies on conventional angiography, ultrasonography, contrast-enhanced CT scan, and MR angiography. The most sensitive imaging modality is contrast-enhanced abdominal CT scan with high quality 3D reconstructions allowing the visualization of the dissected membrane [12]. Possible diagnostic clues in this setting are the presence of an intimal flap associated with an eccentric mural thrombus in the celiac lumen [2, 6]. Interestingly, in case of US imaging, the diagnosis relies on the finding of an abnormal flow as the site of dissection is rarely seen [13].

The treatment of SCIAD is not anyway easier than its diagnosis as it constitutes a major challenge for physicians with the controversial reports. In view of the sparse data, the management plan of SCIAD is extrapolated from the approach to other more common locations of arterial dissections. It mainly relies on the hemodynamic status and the consequent visceral suffering of the patient. In those without visceral involvement and stable blood pressure, there is a trend to strictly control the blood pressure and to prevent visceral thromboembolism showering with a therapeutic anticoagulation. On the other hand, in patients with signs of ischemia and uncontrollable blood pressure, surgical or endovascular interventions are recommended with the latter in patients with high surgical risks [11].

## 4. Conclusion

The natural course of celiac artery dissection is unpredictable and its management is still controversial and requires a case-by-case analysis. To our knowledge, SICAD reports are sparse in the literature. In this paper, we underline the necessity to maintain a high level of suspicion for arterial dissections in patients with wide pattern of blood pressure variations where early diagnosis and optimal management allow a change in prognosis.
